# Application of additional three-dimensional materials for education in pediatric anatomy

**DOI:** 10.1038/s41598-023-36912-9

**Published:** 2023-06-20

**Authors:** Joong Kee Youn, Han Sang Park, Dayoung Ko, Hee-Beom Yang, Hyun-Young Kim, Hyun Bae Yoon

**Affiliations:** 1 Department of Pediatric Surgery, Seoul National University Hospital, Seoul, Korea; 2 Department of Pediatric Surgery, Seoul National University College of Medicine, 101 Daehak-Ro, Jongro-Gu, Seoul, 03080 Korea; 3 Department of Surgery, Seoul National University Bundang Hospital, Seongnam, Gyounggi Korea; 4 Office of Medical Education, Seoul National University College of Medicine, Seoul, Korea

**Keywords:** Gastrointestinal system, Paediatric research

## Abstract

We conducted this study to investigate the effects of additional education using 3D visualization (3DV) and 3D printing (3DP) after applying 2D images for anatomical education in normal pediatric structures and congenital anomalies. For the production of 3DV and 3DP of the anatomical structures, computed tomography (CT) images of the four topics (the normal upper/lower abdomen, choledochal cyst, and imperforate anus) were used. Anatomical self-education and tests were administered to a total of 15 third-year medical students with these modules. Following the tests, surveys were conducted in order to evaluate satisfaction from students. In all four topics, there were significant increases in the test results with additional education with 3DV after initial self-study with CT (*P* < 0.05). The difference in scores was highest for the imperforate anus when 3DV supplemented the self-education. In the survey on the teaching modules, the overall satisfaction scores for 3DV and 3DP were 4.3 and 4.0 out of 5, respectively. When 3DV was added to pediatric abdominal anatomical education, we found an enhancement in understanding of normal structures and congenital anomalies. We can expect the application of 3D materials to become more widely used in anatomical education in various fields.

## Introduction

Recently, 3D printing technology has been applied and utilized in anatomy education of medical students^1–4^. Attempts have been made to replace anatomy education using cadavers by printing not only a single organ but also a whole system, such as the head and neck, thoracic cavity, or blood vessels^5–7^. In addition, it has been recently reported that simulation-based student education using 3D printed objects improves medical understanding^2,8^.

In the education of students on the anatomy of congenital abdominal diseases in children, existing atlases and textbooks of human anatomy have been used as a basis, and in clinical practice education, anatomy is taught through radiologic imaging. However, due to the nature of congenital diseases, it is not easy to improve students' understanding by educating them using only 2D media^9,10^. Therefore, in the field of congenital diseases, studies using 3D printing have been reported for anatomy education on congenital heart disease^4,10–12^. However, few studies have examined the effectiveness of 3D printing in the education of congenital abdominal diseases.

In addition to the existing methods of anatomy education through computed tomography (CT), magnetic resonance imaging (MRI), and contrast studies that have been used in clinical education, we attempted to determine whether additional learning through 3D visualization (3DV) and 3D printing (3DP) is helpful in understanding the anatomical structure of congenital pediatric abdominal diseases. In detail, we aimed to determine the effects of additional learning in topics of normal pediatric abdominal anatomic structures, as well as in abdominal anomalies, specifically choledochal cysts and imperforate anus.

## Methods

### Study protocol

Fifteen third-year medical students practicing in the pediatric surgery department of our center between October 1 and December 31, 2020, participated in this study.

**Figure 1:**
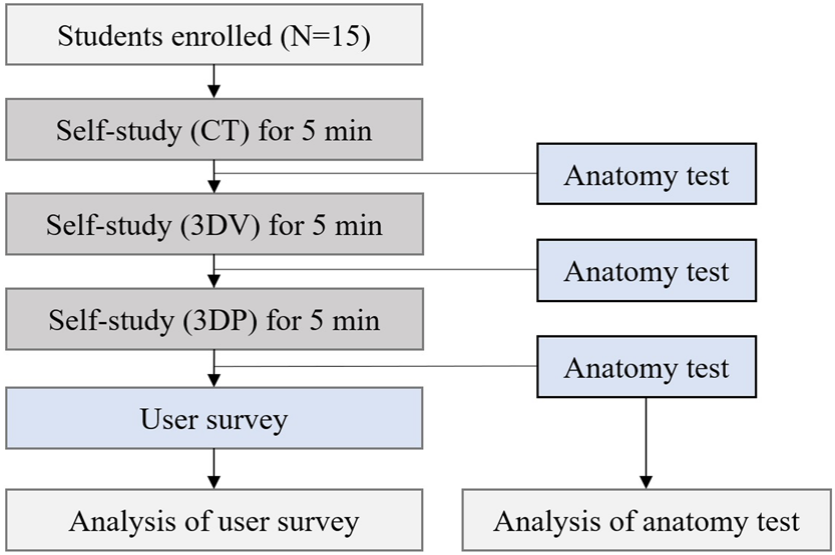
Diagram of the study protocol.

A 1:1 face-to-face test was conducted with 15 students. Each student was tested in four sessions, each of which covered a different anatomical topic (normal upper abdomen, normal lower abdomen, choledochal cyst, and imperforate anus). In each session, self-education was first performed using CT images for 5 min, and an anatomy test was performed as a base score (Supplementary data 1). Next, additional self-education with 3DV was followed by another anatomy test using the same items. Finally, 3DP was used for the last additional self-education step, and the final test was conducted with the same questions (Fig. 1). When all of the above tests for one session were performed, the same process was repeated by moving to the next session. The results of the anatomical tests were converted to a scale of 10 points.

After completing the anatomy tests, each student completed a user survey about the efficiency, authenticity, usefulness, and their overall satisfaction of anatomy education through 3DV and 3DP (Supplementary data 2). The user survey was conducted using a 5-point Likert scale, where respondents were asked to rate their agreement level on a scale of 1 to 5, with 1 indicating 'strongly disagree' and 5 representing 'strongly agree.' This approach was informed by several previous studies^13–15^.

### 3D reconstruction process

For 3D reconstruction of congenital anomalies and normal structures of the upper and lower abdomen, CT images of choledochal cysts (CC), imperforate anus (IA), and normal upper and lower abdomen were obtained from four different patients. Three-dimensional visualizations were produced from the reconstruction process, and 3DP products were made based on 3DV images.

**Figure 2:**
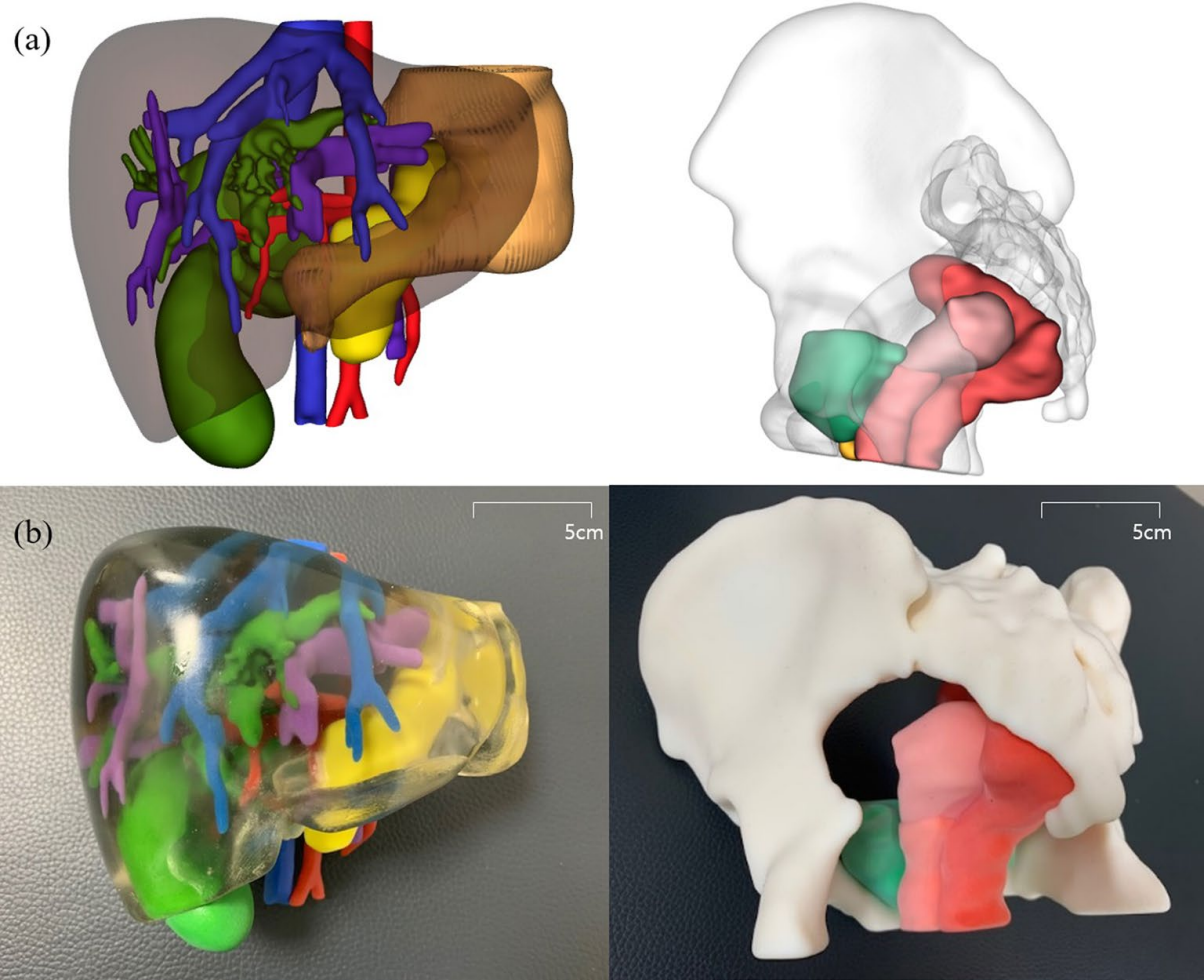
(**a**) 3D visualization and (**b**) 3D printing of choledochal cyst and imperforate anus.

The CT images of these four patients were sent to a 3D printing agency. The agency implemented 3DV using MEDIP software, and this process was done under the confirmation of a pediatric radiologist. The 3DV can be rotated 360° by the user and can be enlarged or reduced in any direction, and the transparency of each organ can be adjusted from 0 to 100% (Fig. 2a). After the 3DV was confirmed, a 3DP model was created on a 1:1 scale (Fig. 2b). Models were printed with acrylonitrile butadiene styrene (ABS) copolymers (400-500 g per case) and resin (800-1200 g per case) by the Polyjet method (J750 [Stratasys; 7665 Commerce Way Eden Prairie, MN 55,344, USA]).

### Statistical analysis

Continuous variables are reported as mean ± standard deviation. IBM SPSS Statistics 20.0 statistical software package (SPSS Inc., Chicago, Illinois, USA) was used for all statistical analyses. The Kruskal-Wallis test was used to confirm that there was a significant difference in the test scores of the three groups (CT, 3DV, and 3DP), and the comparisons between two groups were analyzed using the Mann-Whitney test. Afterwards, according to Bonferroni's method, results were judged to be significant when the *p*-value was < 0.017.

### Ethics approval and recruitment

This study was conducted after obtaining approval from the ethical review board of the Seoul National University Hospital (IRB number: 2003-135-1110), and all methods were performed in accordance with the relevant guidelines and regulations. This study was supported by the SNUH Research Fund (grant number 0420202230). Students’ participation was voluntary, and informed consent was obtained from each student prior to the study.

## Results

**Figure 3:**
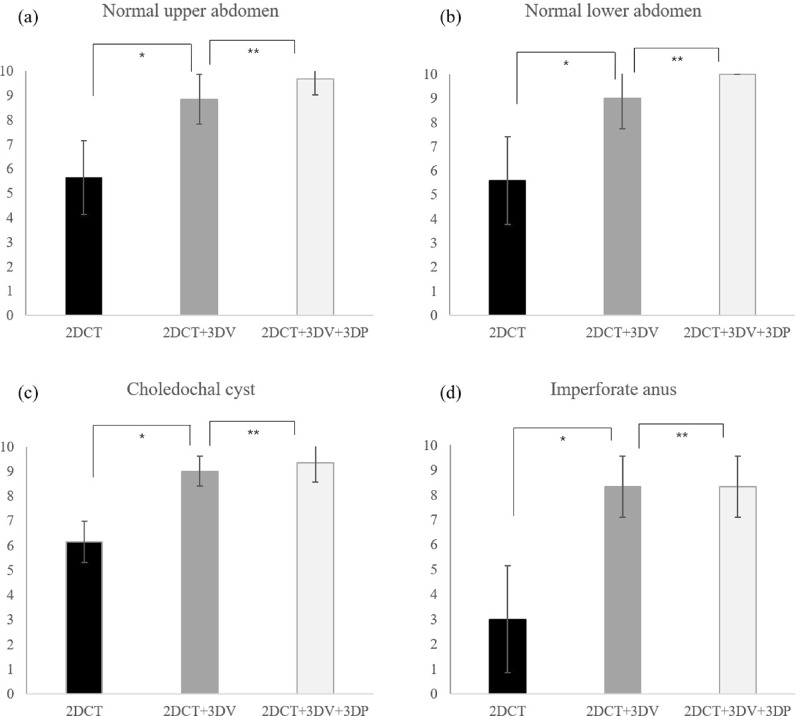
Test scores according to 3D reconstruction models using each set of modules. *Statistically significant, **Statistically insignificant.

When comparing the test results after education using CT images, 3DV, and 3DP, there was a significant difference in all four sessions. When the results after CT training and the results of additional 3DV training were compared, there was a significant difference in the scores in all four sessions. This was also statistically significant when the whole normal structure and the whole congenital disease were compared at once. However, when additional education was provided with 3DP after 3DV education, there were no significant differences in any of the sessions. (Table 1 and Fig. 3).

**Table 1:** Scores after self-study using each set of modules.

	2D CT	2D CT + 3DV	2D CT + 3DV + 3DP	*P* value
*Normal anatomy*				
Upper abdomen	5.6 (± 1.5)	8.9 (± 1.1)	9.7 (± 0.7)	< 0.001
Lower abdomen	5.6 (± 1.8)	9.0 (± 1.3)	10.0 (± 0.0)	< 0.001
*Congenital anomaly*				
Choledochal cyst	6.2 (± 0.8)	9.0 (± 0.6)	9.3 (± 0.8)	< 0.001
Imperforate anus	3.0 (± 2.2)	8.3 (± 1.2)	8.7 (± 1.3)	< 0.001

All *P* values between 2DCT + 3DV and 2DCT were statistically significant.

All *P* values between 2DCT + 3DV + 3DP and 2DCT + 3DV were statistically insignificant.

*CT* Computed tomography, *3DV* 3D visualization, *3DP* 3D printing.

When comparing the difference in score between CT training and additional 3DV training, four topics showed a statistically significant difference, with the largest difference in IA. The difference in scores between 3DV and additional 3DP training was not statistically different in any of the four sessions (Table 2).

**Table 2:** Comparison of test score differences between study sets of modules.

Test score difference	Normal anatomy		Congenital anomaly		*P* value
	Upper abdomen	Lower abdomen	CC	IA	
2DCT / 2DCT + 3DV	3.2 (± 1.3)	3.4 (± 1.9)	2.9 (± 0.7)	5.3 (± 2.1)	< 0.001
2DCT + 3DV / 2D CT + 3DV + 3DP	0.8 (± 0.5)	1.0 (± 1.3)	0.3 (± 0.8)	0.3 (± 0.9)	0.106

*CC* Choledochal cyst, *IA* Imperforate anus, *CT* Computed tomography, *3DV* 3D visualization, *3DP* 3D printing.

From the user survey questionnaire, the efficiency, authenticity, usefulness, and overall satisfaction of anatomy education through 3DV and 3DP were higher than 4.0 and were not statistically different from each other (Table 3).

**Table 3:** User survey on the study modules.

	3D visualization	3D printing	*P* value
Efficiency (3 items)	4.6	3.5	0.507
Authenticity (2 items)	3.2	3.4	0.683
Usefulness (3/4 items)	4.6	4.3	0.858
Overall satisfaction (3 items)	4.5	4.5	1.000
Total	4.3	4.1	0.548

Scale: 1 (Strongly disagree) to 5 (Strongly agree).

## Discussion

Pediatric anatomy is more difficult to understand than that of adults and, particularly in patients with congenital diseases, it is even more difficult to understand at the student level^9,10,16^. When treating patients with congenital pediatric diseases in the clinic, the first step is to perform imaging studies and then gradually understand the specific anatomical differences of these congenital disease patients.

Through this anatomical structure education, 3D printing has been used in various ways, and the results of using 3D printing for education in almost all anatomical structures, such as the liver, lungs, spine, brain, and head and neck, have been actively reported in the education field^17–21^. Efforts have been made to apply 3D printing not only to education in normal structures but also to diseases, complex structures, and even congenital anomalies^22–24^.

Among congenital diseases, numerous studies related to education on congenital anomalies of the heart have been published^4,10,12,25^. A French study conducted 3D printing and compared the educational effects of 3D printed models with 2D images of atrial septal defect, ventricular septal defect, coarctation of the aorta, and tetralogy of Fallot for 347 students. It was observed that education using 3D printing not only improved objective knowledge but also student satisfaction^25^. Similar studies have been conducted with students and residents of various scales, and the similarity of the results was confirmed^4,26^. There are also reports that 3D printing can be used to improve surgical techniques for congenital heart defects beyond simple structural education^27^.

Similar results have been reported in education on congenital head and neck anomalies other than congenital heart disease. For example, in studies related to maxillofacial deformities such as cleft lip and palate or craniosynostosis, 3D reconstruction and 3D printing were performed for student education, and their educational effect was confirmed to be positive^28,29^.

However, few studies have been published on education using 3D materials for congenital abdominal diseases such as choledochal cysts or duodenal atresia. Of course, in education on abdominal organ anatomy, studies on adult abdominal anatomy have shown positive effects of 3D printing. In 2015, Xiangxue et al. studied the effectiveness of 3D visualization images and 3D printing in teaching adult hepatic segment anatomy and reported its effectiveness^30^. Another recent study on anatomical education of the gastrocolic trunk using 3D printing reported good educational results^5^. However, as mentioned above, it is uncommon to use 3D printing for anatomical education on congenital abdominal anomalies in children. Therefore, we tried to determine the effect of additional education with 3D materials after understanding the structures with radiologic imaging such as CT, which has not been previously published.

The results of our study showed that the educational effect improved in all categories when the 3D module training was added after self-education with CT. This is due to an effect of additional education itself; but the fact that 3D teaching materials are used rather than 2D textbooks is also effective. This correlates with previous reports on the effect of 3D materials in education. In the field of medical education, 3D visualization images or printing have the potential to improve students’ understanding of the complex structures of human anatomy^6^.

Through an additional analysis, the educational effect of 3D visualization images was found to be the greatest in IA. IA is not typically diagnosed by conventional CT scans, and the information that can be obtained through CT is limited. In addition, since the pelvic cavity of children is very narrow, it is challenging to determine the anogenital structure using CT; therefore, these limitations can be addressed through 3D visualization images.

In this study, regarding either pediatric normal anatomy or congenital disease anatomy education, both 3DV and 3DP had significant educational effectiveness. However, the effect of additional 3D printing-based education following the 3D visualization-based education was not statistically significant. In the case of 3D visualization technology, it is currently advanced to a high level, and can provide familiar and high-quality information to students who consistently use tablets or high-performance personal computers. Furthermore, in the case of the 3D visualization program used in this study, the ability to zoom-in and -out and to control opacity for each organ would have been a high advantage for education. In contrast, there is still room for improvement in 3D printing. There are limitations in expressing various colors within a single organ, as well as imperfections in expressing small organs or fine structural relationships^31^. Variety of object texture that can be achieved through 3D printing is also subject to limitations, and research efforts to overcome these limitations are being reported^32–34^. For instance, a recent study successfully demonstrated the printing of multi-material structures using a single 3D printer, incorporating both soft and hard materials^35^. If these attempts become more widespread, it is expected that obstacles in 3D printing can be overcome in the near future.

The limitations of this study include the small number of student participants and the fact that only self-education was conducted; there was no separate teaching session. As mentioned above, 3D printed materials can also be considered a limitation of research using this technology, and the fact that there is no way to implement soft tissue in the 3D visualization of CT images is another limitation.

Future research could be divided into two main categories. One would examine the effect of congenital anatomy education using only 2D CT versus 3D printing, not serial education as used in this study. Another category would conduct surgical training of congenital anomalies using 3D printed models and check its effectiveness. The target diseases would include esophageal atresia, duodenal atresia, and choledochal cysts.

## Conclusions

We found additional education using 3D modules after self-education with 2D CT was effective in pediatric normal anatomy and congenital anomalies. Considering the time and cost of producing 3D printed models, 3D printing-based education is not yet superior to 3D visualization-based education in pediatric abdominal anatomy education. However, the 3D printing model has an irreplaceable advantage in that students can directly touch and feel it compared to the 3D visualization model. As technology improves, if each organ can be implemented similarly to the actual texture and even reproduce the fine structural relationships, by replacing cadaveric education, the limitations of existing anatomy education will be overcome with 3D materials.

## Supplementary Information

Supplementary Information.

## Data Availability

The datasets used and/or analyzed during the current study available from the corresponding author on reasonable request.
