# Supplementary material for: Application of additional three-dimensional materials for education in pediatric anatomy
Source: Sci Rep. 2023 Jun 20;13:9973. doi: 10.1038/s41598-023-36912-9 (PMC10282057; doi:10.1038/s41598-023-36912-9)
Supplement: Supplementary file 1 — Supplementary Information. [file 41598_2023_36912_MOESM1_ESM.docx]

Supplementary data 1. Anatomy test items for each session


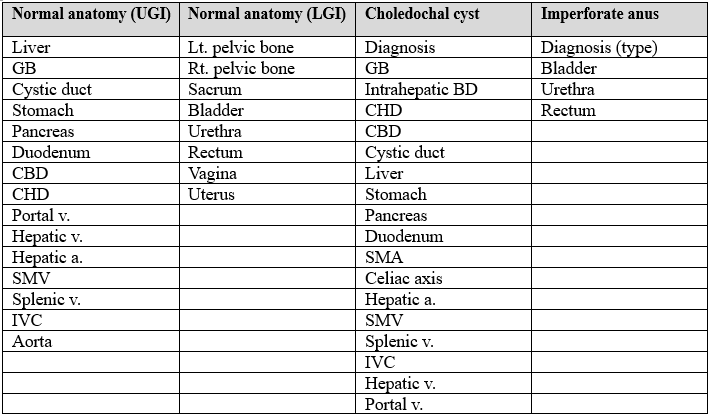


Supplementary data 2. User survey items for (a) 3D visualization and (b) 3D printing

(a)

| **Items** | **Strongly disagree** | **Disagree** | **Neutral** | **Agree** | **Strongly agree** |
| --- | --- | --- | --- | --- | --- |
| Efficiency | | | | | |
| 1. 3D visualization module was easy to handle. |  |  |  |  |  |
| 2. Accessibility to 3D visualization module was better than that of 2D CT. |  |  |  |  |  |
| 3. Accessibility to 3D visualization module was better than that of 3D printing. |  |  |  |  |  |
| Authenticity | | | | | |
| 4. 3D visualization module presented authentic features. |  |  |  |  |  |
| 5. 3D visualization presented integral anatomical features. |  |  |  |  |  |
| Usefulness | | | | | |
| 6. 3D visualization module aroused my interests in anatomy. |  |  |  |  |  |
| 7. 3D visualization module aided in spatial comprehension. |  |  |  |  |  |
| 8. 3D visualization gave me more information than 2D CT for anatomical education. |  |  |  |  |  |
| Overall satisfaction | | | | | |
| 9. I was satisfied with studying abdominal anatomy with 3D visualization. |  |  |  |  |  |
| 10. I’m glad to participate in more education with 3D visualization modules. |  |  |  |  |  |
| 11. Please speak freely about anatomy education with 3D visualization. |  | | | | |

(b)

| **Items** | **Strongly disagree** | **Disagree** | **Neutral** | **Agree** | **Strongly agree** |
| --- | --- | --- | --- | --- | --- |
| Efficiency | | | | | |
| 1. 3D printing module was easy to handle. |  |  |  |  |  |
| 2. Accessibility to 3D printing module was better than that of 2D CT. |  |  |  |  |  |
| 3. Accessibility to 3D printing module was better than that of 3D visualization. |  |  |  |  |  |
| Authenticity | | | | | |
| 4. 3D printing module presented authentic features. |  |  |  |  |  |
| 5. 3D printing presented integral anatomical features. |  |  |  |  |  |
| Usefulness | | | | | |
| 6. 3D printing module aroused my interests in anatomy. |  |  |  |  |  |
| 7. 3D visualization module aided in spatial comprehension. |  |  |  |  |  |
| 8. 3D printing gave me more information than 2D CT for anatomical education. |  |  |  |  |  |
| 9. 3D printing gave me more information than 3D visualization for anatomical education. |  |  |  |  |  |
| Overall satisfaction | | | | | |
| 10. I was satisfied with studying abdominal anatomy with 3D printing. |  |  |  |  |  |
| 11. I’m glad to participate in more education with 3D printing. |  |  |  |  |  |
| 12. Please speak freely about anatomy education with 3D printing. |  | | | | |
| 13. The whole study process utilizing 2D CT, 3D visualization, 3D printing was satisfactory. |  |  |  |  |  |
